# Offering an app to book cervical screening appointments: A service evaluation

**DOI:** 10.1177/0969141319871312

**Published:** 2019-09-09

**Authors:** Mairead Ryan, Laura Marlow, Alice Forster, Josephine Ruwende, Jo Waller

**Affiliations:** 1 Department of Behavioural Science and Health, UCL, London, UK; 2 NHS England (London Region)/Public Health England, London, UK

**Keywords:** Cancer screening, digital health, text message reminders, screening uptake, primary care, behavioural interventions

## Abstract

**Objective:**

To assess the feasibility of offering women who are overdue for cervical screening the use of a smartphone app to book their appointment.

**Methods:**

Women who were at least six months overdue for cervical screening in three general practice surgeries in a deprived East London borough were identified from practice records. Staff sent batches of text messages informing women that they were overdue for screening, and inviting them to download an app to book their appointment.

**Results:**

Across the three practices, 2632 eligible women were identified. Valid mobile phone numbers were available for 1465 women. One woman had opted out of receiving text messages, so messages were sent to 1464 women. Of these, 158 (11%) booked a screening appointment within five months. The majority of these women booked without using the app (72%; 113/158); just over a quarter booked via the app (28%; 45/158).

**Conclusions:**

Just over 10% of cervical screening non-attenders booked an appointment in response to a text message with a link to a downloadable app; however, only one in four of these women booked using the app. This suggests that the text message reminder was likely to have been the key ‘active ingredient’ for most women, rather than the app itself. Future research could explore the optimal message for a text reminder in this context and evaluate the inclusion of a link to existing online booking systems.

## Introduction

The National Health Service (NHS) cervical screening programme aims to reduce cervical cancer incidence and mortality by identifying and treating precancerous lesions. The impact of screening on cervical cancer mortality has been widely acknowledged^1^; however, the success of any screening programme is dependent on high coverage. Coverage for cervical screening, defined as ‘the percentage of women in a population eligible for screening at a given point in time who were screened adequately within a specified period’ ( NHS Cervical Screening Programme,^2^ p.6), is sub-optimal in England, and continues to fall year-on-year, particularly among younger women.^2^ As of 31 March 2018, overall coverage was 71.4%,^2^ which is considerably below the national target of 80%. Age-appropriate coverage among women aged 25–49 fell from 73.7% in 2011 to 69.1% in 2018, and London is the area with the lowest overall coverage in England (64.7% coverage for women aged 25–64 in London vs. 71.4% in England).^2^

While reasons for non-attendance are complex,^3^ practical issues, such as appointment scheduling difficulties, have been identified as a central barrier to participation.^4^ A recent survey found that, among women not currently up-to-date with screening, around half intended to take part,^5^ suggesting that ‘nudge’-based interventions to help translate positive intentions into action could have a significant impact. One such approach is to make the process of booking an appointment easier, and to facilitate the immediate translation of intention into behaviour. Mobile phone applications (apps), software programs installed and run locally on smartphones,^6^ offer the potential for this, allowing appointments to be booked outside normal practice opening hours. Sending a text message including a link to download such an app could be a way of facilitating screening uptake in women who are positively inclined to take part but who are currently overdue.

This service evaluation aimed to assess the feasibility of using a primary care focused app, within the context of cervical screening, to make it easier for overdue women to book screening appointments. The service evaluation also sought to establish the feasibility of collecting relevant data to evaluate such an approach.

## Methods

The service evaluation was carried out in three general practices in Tower Hamlets, East London, an area with a deprived and ethnically diverse population^7^ and low cervical screening uptake (60.9% coverage for women aged 25–64).^8^ All eligible women receive a written invitation to attend for screening and an educational leaflet, enabling invitees to make an informed choice about attendance. Where services have not received a test result from the relevant laboratory within 18 weeks of the invitation letter being created, the woman is considered to be ‘overdue’, and a reminder letter is sent. Thereafter, responsibility lies with general practices to remind overdue women to attend. As part of this on-going activity, staff in three general practices identified all women aged 25–64, eligible for cervical screening (i.e. had not had a hysterectomy or opted out of the programme), who were overdue by at least six months. All patients were identified via EMIS, an electronic patient record system. Staff excluded women who were pregnant or less than three months’ post-partum, and those who were within a current screening episode (i.e. had received an invitation recently but had not yet been sent a reminder). Women aged 25–49 who were last screened four or more years ago, and women aged 50–64 who were last screened six or more years ago, were therefore included. Within each practice, details of women identified by the EMIS query were sorted alphabetically, and staff selected a proportion of women to be contacted each week. The number selected was based on the number of appointment slots available for screening within the practice.

The ‘myGP’ app was developed by iPLATO and is included in the NHS apps library. It is free to download and allows patients to book general practice appointments using a smartphone. Patients register by entering their date of birth and mobile number, and then searching for their general practice. Practices included in the service evaluation had existing contracts in place with iPLATO for their text messaging platform. The myGP app was not used or promoted to patients in two of the three practices (Practice 2 and Practice 3); however, one practice (Practice 1) encouraged patients to use the app, by displaying promotional posters in the reception area, and encouraging service users to download the app to book standard appointments. During the service evaluation period, the practices allocated a proportion of their cervical screening appointment slots to the app. Appointments were labelled on the app as ‘SMEAR TEST (WOMEN AGE 25–64 YRS ONLY)’, to deter other app users from booking these nurse appointments.

Practice staff scheduled text messages to be sent to eligible women, via the iPLATO platform, in weekly batches over six weeks. The text message notified women that they were overdue for cervical screening and invited them to download the app to book an appointment. For example, Practice 1 patients were sent the following message: ‘*From Practice 1: Dear [FIRST NAME], your smear test (cervical screen) is overdue, you can book it directly using the myGP app:* * *link to download app provided*’. Text messages were scheduled to be sent at 12:30 pm, which has been found to be the optimal time for app downloads among female users (personal communication from iPLATO). Women who downloaded and registered on the app were able to view, book and cancel available appointments at their general practitioner (GP) practice. Women could also use standard booking methods to make an appointment in the usual way, including phoning the practice, booking using Patient Access (an online booking system) or face-to-face at reception.

We developed a list of outcomes to assess the feasibility of the process of contacting women by text message and inviting them to use the app to book an appointment (see Table 1). These data would be necessary for any future trial to evaluate the impact of offering app-based booking on uptake. One of the aims of the service evaluation was to assess the feasibility of collecting these data. Table 1 shows which data we were and were not able to collect. At the end of the service evaluation period, practice staff sent the NHS Number, booking method used, and service evaluation week number for each patient to iPLATO. iPLATO then extracted the rest of the data (e.g. age, postcode (for Index of Multiple Deprivation; IMD), and information on app use and appointment booking) from the practices’ clinical systems. All data were fully anonymised (including replacement of postcode by IMD score) before being sent to researchers for analysis. Practices were compensated for participation and were reimbursed for text message credit spent on the messaging platform.

**Table 1.** Outcomes of interest for each eligible woman.

Measured outcomes
Age and IMD^a^ decile
Valid mobile phone number registered with the GP practice^b^
Previously opted out of receiving text-messages from GP practice
Sent a text message
Successfully booked a screening appointment via the app
Booked a screening appointment using any other method
Outcomes of interest for which we were unable to collect data
Opted out of receiving text-messages after receiving the text-message
Downloaded the app during the service evaluation period
Registered their details on the app
Tried unsuccessfully to book a screening appointment via the app
Attended screening

IMD: Index of Multiple Deprivation; GP: general practitioner.

a A deprivation score derived from residential post code.

b A phone number was considered valid if the text message was successfully delivered.

Ethical approval was not needed as the service evaluation did not involve randomisation or the use of identifiable data, and it did not involve changing patient care from accepted standards.

All analyses were conducted using IBM SPSS version 22. We report descriptive data on the outcomes that were collected (see Table 1). These outcomes were stratified by age, IMD decile and general practice.

## Results

While the outcomes shown in Table 1 were sought for each patient, iPLATO were unable to extract all data as intended. The table shows which data iPLATO were able to extract. Across the three practices, 2632 eligible women were identified.

Table 2 shows the data collected for each outcome of interest. Phone numbers were available for 59% (1544/2632) of women identified, but this ranged widely, from 33% at Practice 1 to 98% at Practice 3. Practice 1 did not have the capacity to contact all eligible women identified from the EMIS search query. Thus, the small percentage of phone numbers presented in Table 2 does not reflect the true percentage of phone numbers registered at the practice. Nevertheless, Table 2 highlights that there may be large differences between practices in the proportion of women with registered mobile phone numbers. The majority of registered phone numbers were valid (95%; 1465/1544), and very few women had opted out of receiving text messages from their practice (n = 1). Validity of the numbers was inferred from successful delivery of the text message. Where the message was undeliverable, the number was recorded as invalid.

**Table 2.** Outcomes overall and by GP.

N at each stage of the process	All	Practice 1	Practice 2	Practice 3
Eligible women identified from EMIS (n)	2632	1017	712	903
Available phone numbers (n)	1544	335	700	509
Valid phone numbers (n)	1465	334	632	499
Opted out of receiving texts (n)	1	0	1	0
Sent a text-message (n)	1464	334	631	499
Booked a screening appointment (n)	158	49	54	55
Booked using standard (non-app) methods (n)	113	29	42	42
Booked via the app (n)	45	20	12	13
Percentages				
% eligible women with phone numbers available	58.7	32.9	98.3	56.4
% available phone numbers that were valid	94.9	99.7	90.3	98.0
% women sent a text who booked an appointment	10.8	14.7	8.6	11.0
% women booking who used standard (non-app) methods	71.5	59.2	77.8	76.4
% women booking who booked via the app	28.5	40.8	22.2	23.6

A total of 158 women booked a screening appointment during the study period, which represents 6% of eligible women (158/2632) or 11% of women with valid phone numbers who were sent a text message (158/1464). The majority of these women booked their screening appointment using standard booking options (72%; 113/158), with 45 (28%; 45/158) booking via the app.

Table 3 shows the demographic characteristics of the women with valid mobile numbers, those who booked a screening appointment, and those who booked using the app. The mean age of the sample was 37.3 years (standard deviation (SD): 10.3). Use of the app appeared to be slightly higher in younger women, with 4.4% (95% CI: 3.1–6.1; n = 35) of women aged 25–34 booking using the app, compared with 2.5% (95% CI: 1.2–4.8; n = 9) of women aged 35–44 and only one woman (<1%) aged over 44. The mean age of those using the app to book an appointment was 32.5 (SD: 6.0). A higher proportion of women from Practice 1 booked using the app (6.0%; 95% CI: 3.7–9.1)) compared with Practice 2 (1.9%; 95% CI: 1.0–3.3)) or Practice 3 (2.6%; 95% CI: 1.4–4.4). This may partly be explained by advertising of the app within Practice 1.

**Table 3.** Sample characteristics of women identified as eligible and sent a text-message, those who booked appointments and those who booked via the app (n = 1464).

	All with valid phone numbers	Booked an appointment		Booked via the app	
	N (column %)	N	Row % (95% CI)	N	Row % (95% CI)
All	1464 (100)	158	10.8 (9.2–12.5)	45	3.1 (2.3–4.1)
Age (years)					
25–34	790 (54.0)	93	11.8 (9.6–14.2)	35	4.4 (3.1–6.1)
35–44	354 (24.2)	46	13.0 (9.7–16.9)	9	2.5 (1.2–4.8)
45–54	166 (11.3)	11	6.6 (3.4–11.5)	–	–
55–64	154 (10.5)	8	5.2 (2.3–10.0)	1	0.6 (0.0–3.6)
Area-level deprivation (IMD decile)					
1 (most deprived)	297 (20.3)	30	10.1 (6.9–14.1)	7	2.4 (1.0–4.8)
2	749 (51.2)	84	11.2 (9.0–13.7)	23	3.1 (2.0–4.6)
3	359 (24.5)	37	10.3 (7.4–13.9)	13	3.6 (1.9–6.1)
4	28 (1.9)	2	7.1 (0.9–23.5)	–	–
5 and 6^a^	3 (0.2)	–	–	–	–
Missing	28 (1.9)	5	17.9 (6.1–36.9)	2	7.1 (0.9–23.5)
GP practice					
Practice 1	334 (22.8)	49	14.7 (11.1–18.9)	20	6.0 (3.7–9.1)
Practice 2	631 (43.1)	54	8.6 (6.5–11.0)	12	1.9 (1.0–3.3)
Practice 3	499 (34.1)	55	11.0 (8.4–14.1)	13	2.6 (1.4–4.4)

IMD: Index of Multiple Deprivation; CI: confidence interval; GP: general practitioner.

a No women were from deciles 7–10 (i.e. the least deprived deciles).

## Discussion

This service evaluation aimed to assess the feasibility of offering app-based booking for cervical screening in three GP practices in East London, and of collecting relevant data to evaluate such an approach. Of 1464 women who were sent a text message, 158 women (11%) booked an appointment within a five-month period. The text encouraged some women to book using the app, but the majority (72%; 113/158) booked using standard (non-app) methods.

To our knowledge, there are no research studies examining the efficacy of app-based booking, or previous examples of where this has been used in routine practice^9^ with which to compare our findings; however, previous research has examined the use of text invitations and reminders for cancer screening programmes in the UK and elsewhere (see Uy et al.^10^ for a systematic review). For example, Huf et al.^11^ examined the impact of a text message reminder to increase uptake in cervical screening, and reported a 4% increase in uptake among individuals who received a GP endorsed text message reminder compared with those who did not. Similarly, in the context of colorectal cancer screening, Hirst et al.^12^ reported a 5.6% increase in uptake among individuals who received a text message reminder compared with those who did not. In the absence of a control group, it is not possible to make comparisons between our findings and these studies, as at least some of the women who booked screening appointments may have done so even without the text message intervention. For example, Kitchener et al.^13^ found that uptake of screening in women following their first invitation continued to increase in the absence of interventions over an 18-month period. Furthermore, unlike the samples included in the aforementioned studies, women included in this service evaluation were all at least six months overdue for screening, so at least some of them would probably be women who have decided not to be screened.

Encouraging use of new apps is difficult. Ofcom (the UK regulator for communications services) found that a large proportion of adults had not used any new apps or websites (39%) within the previous month.^14^ A similar proportion (38%) reported having used ‘maybe one or two’ new apps or websites, and a smaller proportion reported having used ‘lots of’ new apps or websites (23%). Ofcom also found that adults in the lowest social grades were less likely to report using new apps or websites within the previous month (50% vs. 39% in higher social grades). Monitoring the impact of such interventions by socioeconomic background will be essential to avoid widening existing social inequalities.

Although statistical comparisons were not carried out, our findings suggest that booking through the app may be more acceptable to younger women, with 4% of 25–34 year olds using the app compared with >1% of 45–64 year olds. This is in line with the study by Ryan et al.,^15^ which found that younger women were more likely to report that they would use app-based booking than older women. Other research studies^16–18^ suggest that younger women are more likely to be overdue for screening for practical reasons, such as appointment booking, whereas older women are more likely to be overdue as a result of low perceived risk or emotional barriers (which we would not expect to overcome with reminders/alternative booking options).

Given that interviews with the overdue population were not conducted as part of the service evaluation, and limited data regarding background characteristics were extracted, it is not possible to make causal inferences about reasons for uptake or barriers to app use within this cohort. Nevertheless, finding that the majority of women did not book their appointment using the app, in conjunction with the findings from Ryan et al.,^15^ suggests that the offer of an app within the context of cervical screening may be less effective than other forms of online booking. Given the apparent reluctance by adults to engage with new apps,^14^ highlighting other forms of online booking already familiar to the target cohort (e.g. Patient Access) may be more appropriate, at least until a single app is more widely used by patients within the NHS. As online services are not currently outlined as a booking option within the NHS cervical screening invitation letter, practices may consider signposting screening invitees to such services. In addition, given the consistent evidence that timed appointments can increase uptake of screening compared with open invitations for all invited women,^19,20^ and non-attenders specifically,^21^ this is another approach that warrants future research. Text messages could be used by primary care providers to offer timed screening appointments, with the option of changing the appointment to a more convenient time.

This service evaluation has several limitations. Firstly, we were not able to ascertain whether women who booked screening appointments actually attended. This would be vital information to collect if the intervention were to be formally trialled. We had limited information on women’s engagement with the app, and were not able to collect data on how many women read the text message, tried to download the app or actually downloaded it in response to the text message. This makes it difficult to understand why more women did not book appointments via the app. Future qualitative research would be useful in addressing this. Finally, we were unable to record the times and dates on which appointments were offered and booked. This may be important data for researchers to collect, as preferences for appointment times for cervical screening have been found to differ by age and deprivation.^22^

## Conclusions

Just over 10% of cervical screening non-attenders booked an appointment in response to a text message with a link to a downloadable app. Only one in four of these women booked using the app. This suggests that the text message reminder was likely to have been the key ‘active ingredient’ for most women, rather than the app itself. The success of any digital intervention to increase uptake for cervical screening may be influenced by many factors, including appointment availability, time of scheduled invitation, patient preferences and competencies, age and socio-economic status. Future research should explore the relative impact of individual intervention components (e.g. booking options or appointment availability), as well as the combination of components and dosage (e.g. number of text reminders) most likely to be effective for various targeted groups of the screening population. Digital interventions aimed at increasing uptake should assess differential effectiveness across population subgroups, to avoid increasing existing socioeconomic inequalities in uptake of cervical screening.

## References

[1] Landy R Pesola F Castanon A , et al. Impact of cervical screening on cervical cancer mortality: estimation using stage-specific results from a nested case-control study. Br J Cancer 2016; 115: 1140–1146. 27632376 10.1038/bjc.2016.290 PMC5117785

[2] NHS Cervical Screening Programme, England 2017–18. National Statistics, 2018. https://files.digital.nhs.uk/B1/66FF72/nhs-cerv-scre-prog-eng-2017-18-report.pdf (accessed 27 August 2019).

[3] Chorley AJ Marlow LA Forster AS , et al. Experiences of cervical screening and barriers to participation in the context of an organised programme: a systematic review and thematic synthesis. Psycho-Oncology 2017; 26: 161–172. 27072589 10.1002/pon.4126 PMC5324630

[4] Waller J Jackowska M Marlow L , et al. Exploring age differences in reasons for nonattendance for cervical screening: a qualitative study. BJOG 2012; 119: 26–32. 21668764 10.1111/j.1471-0528.2011.03030.x

[5] Marlow LAV Chorley AJ Haddrell J , et al. Understanding the heterogeneity of cervical cancer screening non-participants: data from a national sample of British women. Eur J Cancer 2017; 80: 30–38. 28535495 10.1016/j.ejca.2017.04.017 PMC5489076

[6] Heffner JL Vilardaga R Mercer LD , et al. Feature-level analysis of a novel smartphone application for smoking cessation. Am J Drug Alcohol Abuse 2015; 41: 68–73. 25397860 10.3109/00952990.2014.977486 PMC4410684

[7] Healthy Life Expectancy in Tower Hamlets. Annual Public Health Report of the Director of Public Health. 2018. https://www.towerhamlets.gov.uk/Documents/Public-Health/Tower_Hamlets_Public_Health_Report_2018.pdf (accessed 27 August 2019).

[8] PHE Fingertips. ‘Females, 25-64, attending cervical screening within target period (3.5 or 5.5 year coverage,%)’ 2017/18. https://fingertips.phe.org.uk/search/cervical%20screening (accessed 27 August 2019).

[9] Jo's Trust Cervical screening in the spotlight: One year on. 2018. https://www.jostrust.org.uk/sites/default/files/cervical_screening_in_the_spotlight_2018_final.pdf (accessed 27 August 2019).

[10] Uy C Lopez J Trinh-Shevrin C , et al. Text messaging interventions on cancer screening rates: a systematic review. J Med Internet Res 2017; 19: e296. 28838885 10.2196/jmir.7893 PMC5590008

[11] Huf S King D Kerrison R , et al. Behavioural text message reminders to improve participation in cervical screening: a randomised controlled trial. Lancet 2017; 390: S46.

[12] Hirst Y Skrobanski H Kerrison RS , et al. Text-message Reminders in Colorectal Cancer Screening (TRICCS): a randomised controlled trial. Br J Cancer 2017; 116: 1408–1414. 28441381 10.1038/bjc.2017.117 PMC5520096

[13] Kitchener HC Gittins M Rivero-Arias O , et al. A cluster randomised trial of strategies to increase cervical screening uptake at first invitation (STRATEGIC). Health Technol Assess 2016; 20: 1–138. 10.3310/hta20680 PMC5046076 27632816

[14] Ofcom. Adults’ Media Use and Attitudes Report. 2018. https://www.ofcom.org.uk/__data/assets/pdf_file/0011/113222/Adults-Media-Use-and-Attitudes-Report-2018.pdf (accessed 26 August 2019).

[15] Ryan M, Waller J, and Marlow LA. Could changing invitation and booking processes help women translate their cervical screening intentions into action? A population-based survey of women's preferences in Great Britain. *BMJ Open* 2019; 9: e028134. 10.1136/bmjopen-2018-028134 PMC6629419 31300499

[16] Waller J Bartoszek M Marlow L , et al. Barriers to cervical cancer screening attendance in England: a population-based survey. J Med Screen 2009; 16: 199–204. 20054095 10.1258/jms.2009.009073

[17] Marlow LAV Ferrer RA Chorley AJ , et al. Variation in health beliefs across different types of cervical screening non-participants. Prevent Med 2018; 111: 204–209. 10.1016/j.ypmed.2018.03.014 PMC5945192 29550302

[18] Bennett KF Waller J Chorley AJ , et al. Barriers to cervical screening and interest in self-sampling among women who actively decline screening. J Med Screen 2018; 25: 211–217. 29649936 10.1177/0969141318767471 PMC6262593

[19] Everett T Bryant A Griffin MF , et al. Interventions targeted at women to encourage the uptake of cervical screening. Cochrane Database Syst Rev 2011; 5: CD002834. 10.1002/14651858.CD002834.pub2 PMC4163962 21563135

[20] Camilloni L Ferroni E Cendales BJ , et al. Methods to increase participation in organised screening programs: a systematic review. BMC Public Health 2013; 13: 464. 10.1186/1471-2458-13-464 PMC3686655 23663511

[21] Lonnberg S Andreassen T Engesaeter B , et al. Impact of scheduled appointments on cervical screening participation in Norway: a randomised intervention. BMJ Open 2016; 6: e013728. 10.1136/bmjopen-2016-013728 PMC5128903 28186949

[22] Olowokure B Caswell M Duggal HV. What women want: convenient appointment times for cervical screening tests. Eur J Cancer Care 2006; 15: 489–492. 10.1111/j.1365-2354.2006.00703.x 17177908

